# Progranulin and Vaspin as Potential Novel Markers in the Etiology of Type 1 Diabetes in Children

**DOI:** 10.3390/medicina60071165

**Published:** 2024-07-19

**Authors:** Katarzyna Jakubek-Kipa, Sabina Galiniak, Artur Mazur

**Affiliations:** 1 Department of Pediatrics, Institute of Medical Sciences, Medical College of Rzeszów University, Warzywna 1a, 35-310 Rzeszów, Poland; drmazur@poczta.onet.pl; 2 Department of Medical Chemistry and Metabolomics, Institute of Medical Sciences, Medical College of Rzeszów University, Warzywna 1a, 35-310 Rzeszów, Poland; sgaliniak@ur.edu.pl

**Keywords:** diabetes, progranulin, vaspin

## Abstract

*Background and Objectives:* Diabetes is a significant health problem, prompting the search for new therapeutic strategies. Recently, researchers have focused on identifying novel markers for the progression of this condition. It is well established that adipokines, such as progranulin and vaspin, play crucial roles in regulating lipid and carbohydrate metabolism. *Materials and Methods*: This single-center cross-sectional study aimed to assess serum progranulin and vaspin levels in 80 children diagnosed with type 1 diabetes (T1D) and to examine their correlation with body mass index (BMI), glycated hemoglobin, and lipid profile. The cohort included 40 children newly diagnosed with diabetes, 40 children with long-term diabetes (20 well-controlled and 20 poorly controlled), and 14 non-diabetic children as a control group. Progranulin and vaspin levels were determined using a sandwich enzyme-linked immunosorbent assay. *Results*: There were no significant differences in the progranulin and vaspin concentrations in the studied groups (*p* = 0.246 and *p* = 0.095, respectively). No statistically significant differences were noted in the levels of both adipokines among boys and girls within the T1D, well-controlled T1D, and poorly controlled T1D groups. We did not find any differences in the progranulin and vaspin levels among all children with T1D and healthy controls when divided based on BMI percentiles. A negative correlation was observed between progranulin concentration and the age of children in the T1D, well-controlled T1D, and healthy groups. Furthermore, progranulin correlated negatively with BMI among children with T1D. In contrast, vaspin concentration correlated positively with age among healthy children. *Conclusions*: Our study provides novel insights into the status of progranulin and vaspin among pediatric participants with varying levels of type 1 diabetes control. However, further research involving larger patient cohorts and different stages of sexual maturation is warranted.

## 1. Introduction

Diabetes is the first disease that the Organization of the United Nations recognized as an epidemic of the 21st century. In Poland, approximately 3 million Poles suffer from it [1]. In children, the dominant type is autoimmune diabetes, and the incidence of this disease is high [2]. However, data from recent years have suggested that the incidence of type I diabetes (T1D) in children, after a previous rapid increase, has now stabilized. Recent data published by researchers from Ireland show that the incidence in this country has increased again [3]. Moreover, recently, an increase in new cases of T1D and diabetic ketoacidosis has been reported, particularly since the COVID-19 pandemic [4,5].

Due to the fact that this disease is a significant health problem, new ideas are being sought to develop therapeutic strategies for this disease. It is known that adipokines influence the regulation of carbohydrate metabolism. Adipokines play a role in the pathogenesis of diabetes and also its complications [6]. Progranulin is a cysteine-rich secreted protein expressed in epithelial cells, immune cells, neurons, and adipocytes. A recently discovered function of progranulin is its influence on glucose metabolism [7]. There is emerging evidence that progranulin has a protective effect in the development of various autoimmune diseases, including T1D, by regulating signaling pathways [8]. It has also been shown that hyperprogranulinemia is involved in the pathogenesis of obesity-related insulin resistance [9]. Vaspin is a novel adipocytokine that has potential insulin-sensitizing effects. Higher vaspin serum levels were associated with an increased risk of diabetic retinopathy in patients with type 2 diabetes mellitus [10]. The aim of this study was to assess the level of progranulin and vaspin in children with T1D and to correlate serum levels of progranulin and vaspin with body mass index (BMI), glycated hemoglobin (HbA1c), and lipid profile.

## 2. Materials and Methods

### 2.1. Ethical Approval

The study protocol was approved by the Bioethics Committee of the University of Rzeszow in Poland (protocol number: 2018/03/08, date: 8 March 2018). All procedures carried out in studies involving human participants were in accordance with the ethical standards outlined by the institutional and/or national research committee, as well as the principles established in the Declaration of Helsinki of 1964 and its subsequent revisions or equivalent ethical standards. Prior to their participation in the study, written informed consent was obtained from legal guardians and/or children.

### 2.2. Study Subjects

A single-center cross-sectional study was conducted involving a sample of 94 children. Of these, 40 were newly diagnosed with type 1 diabetes (T1D), and 40 had been living with diabetes for over a year. Among the latter group, 20 children had good metabolic control (well-controlled T1D), while the other 20 had poor metabolic control (poorly controlled T1D). The inclusion criteria were consent to participate in the study and aged between 6 and 16 years. All included children were Caucasian with no family history of type 1 or other types of diabetes. The diagnosis of T1D followed the criteria set by the International Society for Pediatric and Adolescent Diabetes. The exclusion criteria were a lack of consent to participate in the study and cooperation with a doctor, abnormal liver function parameters, anemia, taking immunosuppressive drugs and/or steroids, active bacterial or viral infection, and depression. Participants were recruited from the Department of Pediatrics, Pediatric Endocrinology and Diabetology, and the Endocrinology Outpatient Clinic between 2019 and 2021. Progranulin and vaspin levels were measured after the stabilization of the participants’ general condition. Children diagnosed with T1D received intensive insulin therapy using insulin analogs, administered either through insulin pens or continuous subcutaneous insulin infusion with personal insulin pumps. Poor metabolic control was defined by an HbA1c level exceeding 7%. The control group consisted of 14 healthy children who were confirmed to be free of T1D based on their medical history, clinical examination, and biochemical tests.

Children with a BMI greater than the 85th percentile for their sex and age were included in both the diabetic and healthy control groups, as a high BMI was not an exclusion criterion. Participants were then categorized into three BMI percentile groups according to WHO criteria: (1) underweight—less than the 3rd percentile, (2) healthy weight—3rd to less than the 85th percentile, and (3) overweight and obese—85th percentile and above.

### 2.3. Blood Collection and Analysis Procedures

In the morning, after an overnight fasting period, a venous peripheral blood sample of 5 mL was obtained and collected in a standard clotting activator tube. Subsequently, the collected samples were allowed to clot and then centrifuged for 10 min at 1000× *g*, maintaining a temperature of 4 °C, using a centrifuge model 5702 R from Eppendorf AG, Hamburg, Germany. The resulting serum was carefully transferred into cryovials and immediately frozen at −80 °C for subsequent analysis. It is essential to highlight that serum samples were not stored for longer than one month, and thawing was carried out only once at room temperature during the analytical procedures. Additionally, supplementary clinical parameters were retrieved from the individuals’ medical records.

### 2.4. Progranulin and Vaspin Assay

Determination of progranulin was prepared with the use of an enzyme-linked immunosorbent assay (ELISA) (E103, Mediagnost, Reutlingen, Germany). Vaspin was also quantified by ELISA (E106, Mediagnost, Germany). The procedure was prepared according to the manufacturer’s protocol. According to the manufacturer’s specifications, the inter- and intra-assay coefficients of variation were below 10% for both ELISAs. Progranulin was expressed in ng/mL and vaspin in pg/mL. Absorptiometric measurements were performed on a Tecan Infinite 200 PRO multimode reader (Tecan Group Ltd.; Männedorf, Switzerland).

### 2.5. Statistical Analysis

Statistical analyses were performed using the STATISTICA software package (version 13.3, StatSoft Inc., Tulsa, OK, USA). Data were expressed as mean ± standard deviation (SD) with the corresponding range. Since most variables did not adhere to a normal distribution, as confirmed by the Shapiro–Wilk test, non-parametric tests were employed. Specifically, the Kruskal–Wallis analysis of variance (ANOVA) was utilized for multiple comparisons or the Mann–Whitney test was used for comparing two groups. Statistical significance was set at a *p*-value less than 0.05. Pairwise comparisons were evaluated using the Bonferroni correction. The *p*-values given in the tables were adjusted accordingly.

## 3. Results

The characteristics of the study cohort are outlined in Table 1. Children diagnosed with T1D were significantly younger compared to both children with poorly controlled T1D (*p* = 0.047) and healthy counterparts (*p* = 0.003). No statistically significant difference was observed in the mean age between genders within the study cohort. Additionally, children within the poorly controlled T1D subgroup had a statistically longer duration of diabetes treatment (*p* < 0.001). Remarkably, children diagnosed with T1D presented with lower body weight compared to both those in the poorly controlled T1D subgroup (*p* = 0.003) and the control group (*p* = 0.034). Similarly, the BMI of children with T1D was significantly lower than that of children in both the poorly controlled T1D subgroup (*p* < 0.001) and the healthy cohort (*p* = 0.014). Additionally, children within the T1D subgroup exhibited elevated levels of HbA1c compared to those with well-controlled T1D (*p* < 0.001) and healthy counterparts (*p* < 0.001). Conversely, children with well-controlled T1D displayed significantly lower HbA1c levels compared to those in the poorly controlled T1D subgroup (*p* = 0.006).

**Figure 1:**
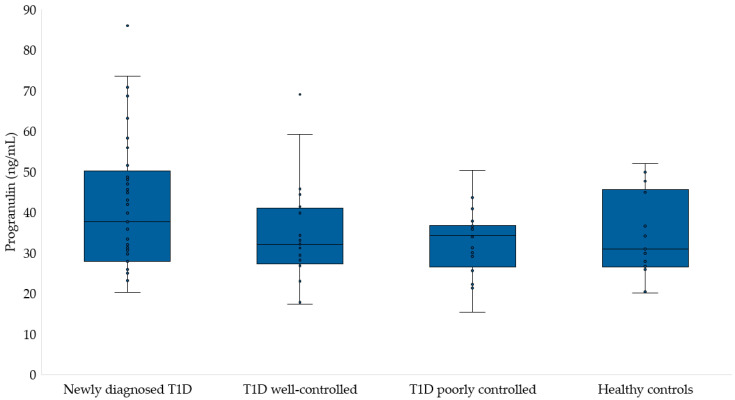
Progranulin concentration in study groups.

**Figure 2:**
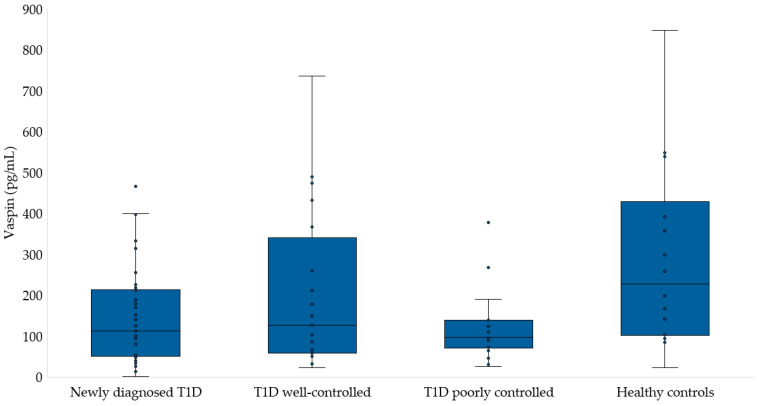
Vaspin concentration in study groups.

Figure 1 and Figure 2 present the levels of progranulin and vaspin in the studied groups. We did not find any difference in the concentration of progranulin among the children. The progranulin concentration in the T1D group was 41.6 ± 16.5 ng/mL, while in well-controlled T1D and poorly controlled T1D children, it was 34.6 ± 12.7 and 32.7 ± 8.4 ng/mL, respectively. Among healthy children, the concentration of this adipokine was 34.1 ± 10.6 ng/mL (*p* = 0.246). Similarly, there were no differences in the vaspin concentrations of the studied groups (T1D: 147.1 ± 116.1 pg/mL, T1D well-controlled: 202.9 ± 196.7, T1D poorly controlled: 123.1 ± 91.9 pg/mL, and healthy children: 289.2 ± 230.3 pg/mL, *p* = 0.095).

Table 2 illustrates the variations in progranulin and vaspin concentrations based on the sex of the study participants. No statistically significant differences were noted in the levels of both adipokines among males and females within the T1D, well-controlled T1D, and poorly controlled T1D groups. However, a noteworthy finding emerged, revealing a significantly elevated level of vaspin in healthy girls compared to healthy boys (*p* < 0.05).

Table 3 and Table 4 present the level of progranulin and vaspin depending on the BMI percentiles. Across all investigated groups, no differences in progranulin concentrations were observed based on the BMI percentiles.

Similarly, no differences in vaspin concentrations were observed across the studied groups based on the children’s BMI percentiles. We noticed that participants with lower BMI (<3rd and between 3rd and 85th percentile) had higher vaspin concentrations than children with higher BMI (>85th percentile). However, this difference was not statistically significant (*p* = 0.083).

The next important step of our study was to determine the correlation between progranulin and vaspin concentrations and participants’ clinical data (Table 5). We observed a negative correlation between progranulin concentration and the age of children in the T1D, well-controlled T1D, and healthy groups. Furthermore, progranulin correlated negatively with BMI among children with T1D. Regarding vaspin, its concentration correlated positively with age among control subjects. No other significant correlations were found in the studied groups.

## 4. Discussion

Although the etiology of many autoimmune diseases is not entirely clear, it is known that uncontrolled inflammatory reactions are the primary cause of their development and progression. Progranulin is a molecule with anti-inflammatory effects that acts on multiple pathways. It serves as an endogenous TNF-α antagonist by competitively binding to its receptor, which induces the differentiation of Tregs—a subpopulation of T lymphocytes with immunosuppressive effects [8]. Progranulin also regulates the expression of interleukin 10, an anti-inflammatory cytokine, and inhibits the release of chemokines from macrophages [11]. Progranulin is also described as an adipokine associated with obesity, insulin resistance, and type 2 diabetes, as well as its pro-inflammatory effects [12,13,14].

In the population of children presented in this study, we did not find any statistically significant differences in progranulin concentration between the study groups. Nevertheless, the mean serum progranulin level was significantly higher in T1D adults with a disease duration of more than 5 years and those with newly diagnosed T1D compared to healthy controls (*p* = 0.009, *p* = 0.032, respectively) in a recent study by Rohoma et al. [15]. Similarly to our results, no significant difference was found in the mean serum progranulin between the two diabetic groups (*p* = 0.883) [15]. There were no significant differences in serum progranulin levels between diabetic subjects with and without metabolic syndrome compared to healthy adult subjects [16]. Moreover, adults with metabolic syndrome had similar blood concentrations of progranulin compared to healthy subjects [17]. The role of progranulin in diabetes and metabolic syndrome has been the subject of investigation due to its potential involvement in insulin resistance and chronic inflammation. However, the lack of significant difference in progranulin levels among the groups studied suggests that progranulin may not be directly linked to the presence of metabolic syndrome in diabetic individuals. This finding indicates that while progranulin may play a role in metabolic processes, it is not a sufficient marker to distinguish between diabetic patients with and without metabolic syndrome. The absence of significant variation in progranulin levels across these groups highlights the complexity of metabolic syndrome, which involves a constellation of factors such as obesity, insulin resistance, hypertension, and dyslipidemia [18]. Therefore, relying solely on progranulin levels for diagnosis or risk assessment of diabetic patients might be insufficient.

In our study, we did not find any differences in the progranulin level among all children with T1D and healthy controls divided based on BMI percentiles. Likewise, the progranulin concentrations did not differ significantly (*p* = 0.795) between obese and normal-weight children [19]. Nevertheless, we noted significantly negative associations between progranulin levels and age in T1D, well-controlled T1D, and healthy children. Moreover, progranulin correlated negatively with BMI among subjects with T1D. However, there are reports that in both adults and children, progranulin levels significantly correlate with BMI [20,21,22]. Progranulin was significantly negatively related to age but not to gender in a recent study [19]. Circulating progranulin significantly correlates with BMI, macrophage infiltration in omental adipose tissue, C-reactive protein serum concentrations, HbA1c values, and total cholesterol in adult patients [23]. Qu et al., in their analysis of subjects with type 2 diabetes, showed a correlation between the level of progranulin and the HbA1c value [24].

Vaspin is a serine protease inhibitor belonging to the serpin group of proteins. It was discovered by Hida et al. in 2005 [25]. Vaspin is expressed in various tissues and organs, including subcutaneous fat, skin, stomach, skeletal muscles, pancreas, and liver [26,27]. Additionally, as a serine protease inhibitor, vaspin has been demonstrated to inhibit the activity of insulin-degrading serine protease, specifically kallikrein 7, in pancreatic islets. This inhibition may potentially prolong the duration of insulin circulation [28]. We did not find any difference in the vaspin concentrations of the studied groups. Elevated vaspin levels in healthy girls could indicate a protective mechanism against metabolic disorders, highlighting the importance of considering sex as a variable in metabolic research and potentially guiding gender-specific therapeutic approaches in the future.

Serum vaspin levels were lower in participants with type 2 diabetes compared to non-diabetic subjects [29]. Surprisingly, the adult subjects with type 2 diabetes exhibited significantly lower serum vaspin levels than the controls (0.62 ± 0.26 vs. 0.83 ± 0.28 ng/mL, respectively, *p* = 0.001) [30]. Recent findings from a study suggest that elevated vaspin levels are associated with an increased risk of type 2 diabetes and reduced gluteofemoral adiposity. These findings position vaspin as a potentially valuable clinical predictor for type 2 diabetes [31].

An interesting issue for us was whether, in participants with T1D, the level of vaspin correlated with the degree of metabolic control, BMI, and lipid metabolism parameters. We noticed that children with lower BMI had higher, but not statistically significant, vaspin concentrations compared to children with higher BMI. Conversely, higher vaspin levels were found in obese children compared to children with normal weight or those who were lean [32,33]. We did not find any other correlations between vaspin levels and clinical parameters in the studied groups with T1D. Research shows that vaspin concentration positively correlates with BMI, impaired glucose tolerance, and insulin resistance in both patients with and without diabetes [29,32,33,34].

While our study offers novel insights into the status of progranulin and vaspin among pediatric participants with varying levels of T1D control, several limitations should be acknowledged. Firstly, the study was conducted at a single center with a relatively small sample size of 80 children. Children were recruited during the COVID-19 pandemic, so obtaining a larger study group was not possible. This limited cohort size might reduce the generalizability of our findings. Subsequent studies should aim to include larger, multi-center cohorts to validate and extend our observations. Secondly, the cross-sectional design of our study provides only a snapshot of adipokine levels at one point in time. Longitudinal studies are needed to explore how progranulin and vaspin levels change over time with disease progression and varying treatment regimens. Thirdly, our study did not account for the potential effects of pubertal status and sexual maturation, which can influence adipokine levels and metabolic parameters. Future research should stratify participants by stages of sexual maturation to better understand these dynamics. Moreover, while we assessed correlations with BMI, HbA1c, and lipid profile, other factors such as diet, physical activity, and genetic background were not considered. Future research should include these variables to control their potential confounding effects. In conclusion, while our study adds to the understanding of progranulin and vaspin in pediatric T1D, further research involving larger, multi-center cohorts, longitudinal designs, and comprehensive metabolic profiling is necessary to elucidate the roles of these adipokines and develop potential therapeutic strategies, especially given the increasing number of obese children in the world.

## 5. Conclusions

In our study, we investigated progranulin and vaspin levels in children with T1D and examined their correlation with clinical parameters. Despite a comprehensive understanding of adipokine influence on carbohydrate metabolism, we observed no significant differences in progranulin and vaspin levels between our study groups. Additionally, there were no significant correlations between these adipokine levels and the clinical parameters of T1D patients.

It is important to note, however, that conflicting data exist regarding the relationship between progranulin levels and obesity, as well as insulin resistance in both children and adults. This highlights the necessity for further research in this area to elucidate these associations.

## Figures and Tables

**Table 1:** Overview of the study participants’ characteristics ^1^.

		Newly Diagnosed T1D	T1D Well-Controlled	T1D Poorly Controlled	Control Group	*p*
n (%)		40 (42.5)	20 (21.3)	20 (21.3)	14 (14.9)	
Sex (girls/boys)		11/29	6/14	10/10	5/9	
Age (years)	mean ± SD	9.5 ± 3.9	11.9 ± 3.9	13.4 ± 3.5 ^AΔ^	14.3 ± 4.8 ^A#^	0.001
	range	2.2–16.1	2.9–17.9	5.1–17.8	3.6–17.6	
Disease duration (months)	mean ± SD	–	27 ± 10.8	79 ± 31.9	–	<0.001
	range	–	11–49	27–152	–	
Weight (kg)	mean ± SD	34.6 ± 16.4	43 ± 16.4	55.4 ± 17.4 ^A#^	54.9 ± 18.7 ^AΔ^	0.001
	range	12–72	13.5–72	20.5–83	15–87	
BMI (kg/m^2^)	mean ± SD	16.8 ± 3.7	18.1 ± 2.7	21.6 ± 3.5 ^A^*	21.2 ± 3.1 ^AΔ^	<0.001
	range	12.5–26.3	14.3–24.2	16.3–28.1	15.0–26.3	
BMI percentiles						
<3rd	n (%)	7 (17.5)	0	0	0	
3rd–85th	n (%)	27 (67.5)	16 (80)	13 (65)	11 (79)	
>85th	n (%)	6 (15)	4 (20)	7 (35)	3 (21)	
HbA1c (%)	mean ± SD	11.8 ± 2.6	6.5 ± 0.5 ^A^*	10.2 ± 2.2 ^B#^	5.3 ± 0.2 ^A^*	<0.001
	range	6.9–17.0	5.23–6.9	8–15.1	4.9–5.6	
TC (mg%)	mean ± SD	178.2 ± 55.7	170.5 ± 28	179 ± 47.4	176.1 ± 11.8	0.929
	range	16–297	129–224	125–292	150–190	
HDL (mg%)	mean ± SD	54.4 ± 30.1	62.7 ± 17.9	58.5 ± 9.3	49.9 ± 7.7	0.007
	range	19–181	29–97	40–78	40–68	
LDL (mg%)	mean ± SD	95.4 ± 44.1	92.1 ± 21	99 ± 35.5	82.1 ± 17.7	0.543
	range	30–206	45–124	44–182	60–115	
TG (mg%)	mean ± SD	297.9 ± 319.6	62.7 ± 17.9 ^A^*	112.7 ± 72.2 ^AΔ^	99.9 ± 27.7	<0.001
	range	53–1800	34–139	41–328	58–156	

^1^ BMI—body mass index; HbA1c—glycated hemoglobin A1; TC—cholesterol; TG—triglycerides; ^A^—when compared to newly diagnosed T1D; ^B^—when compared to T1D well-controlled; ^Δ^—*p* < 0.05, ^#^—*p* < 0.01; *—*p* < 0.001.

**Table 2:** Progranulin and vaspin levels by participant sex.

		Progranulin, ng/mL	Vaspin, pg/mL
Newly diagnosed T1D	Boys	39.8 ± 13.5	133.5 ± 105.8
	Girls	46.6 ± 23	185.2 ± 140.1
	*p*	0.596	0.397
T1D well-controlled	Boys	33.5 ± 12.2	210.6 ± 214.2
	Girls	37.3 ± 14.5	184.9 ± 164.7
	*p*	0.483	0.967
T1D poorly controlled	Boys	32.2 ± 10.3	115.9 ± 72.7
	Girls	33.1 ± 6.4	129.6 ± 109.9
	*p*	0.623	0.967
Control group	Boys	38.9 ± 10.1	134.9 ± 104.9
	Girls	31.4 ± 10.4	374.9 ± 239.9
	*p*	0.124	0.045

**Table 3:** Progranulin levels by BMI percentile.

BMI	Newly Diagnosed T1D	T1D Well-Controlled	T1D Poorly Controlled	Control Group	*p*
<3rd	44.7 ± 17.5	-	-	-	-
3rd–85th	43 ± 17.5	33.3 ± 10.1	33.7 ± 9.3	33.3 ± 11.4	0.226
>85th	33.1 ± 9.1	39.9 ± 21.5	30.7 ± 6.5	37.1 ± 7.5	0.731

**Table 4:** Vaspin levels by BMI percentile.

BMI	Newly Diagnosed T1D	T1D Well-Controlled	T1D Poorly Controlled	Control Group	*p*
<3rd	230.3 ± 167.3	-	-	-	-
3rd–85th	150.1 ± 107.5	219.6 ± 216.1	127.6 ± 100.8	302.9 ± 233.4	0.127
>85th	64 ± 44.4	136.1 ± 63.5	113.3 ± 76.6	239.0 ± 259.9	0.186

**Table 5:** Spearman’s rank correlation coefficients and *p* values for progranulin.

	Newly Diagnosed T1D		T1D Well-Controlled		T1D Poorly Controlled		Control Group	
	*R*	*p*	*R*	*p*	*R*	*p*	*R*	*p*
Age	−0.403	0.010	−0.826	<0.001	−0.090	0.706	−0.747	0.002
BMI	−0.419	0.007	−0.362	0.116	−0.365	0.113	−0.486	0.078
Hb1Ac	0.204	0.207	0.197	0.404	0.069	0.771	0.361	0.204
TC	−0.118	0.469	0.411	0.072	0.229	0.331	−0.342	0.232
HDL	−0.168	0.301	0.194	0.412	0.293	0.211	−0.099	0.735
LDL	−0.120	0.460	0.291	0.214	0.140	0.555	0.129	0.659
TG	0.080	0.624	−0.113	0.636	0.023	0.922	−0.233	0.422

## Data Availability

The data that support the findings of this study are available on request from the corresponding author.
